# Correspondence

**Published:** 1822-03-01

**Authors:** 


### XVII.

### CORRESPONDENCE.

The short anonymous article for the Extra-Limites department is inadmissible. If we have opened a door for authors to *defend* themselves against what they may conceive to be unfair criticism, in this or any other journal, we cannot permit such papers to become *attacks* upon individuals, nor to be couched in other than mild (however energetic) language. *Such* defences would injure us without benefiting them. But we again repeat that every author has free access to the Extra-Limites department of the Journal, provided he avoids all intemperate language and personal allusions—articles which never can be admitted into our pages. The paper and enclosure will be delivered to any one producing the motto or signature.

Several of our literary friends will see that their hints are attended to; and several others, we are sorry to say, may feel disappointed at not finding the analytical articles, which they have so liberally furnished, inserted in the present number. They, however, and the profession at large, will readily perceive that *early* reviews of all works are totally incompatible with that careful *analytical* delineation which is the characteristic feature of this Journal, and to which it mainly owes its success. We could make a tinsel shew of 50 or 100 *critical* articles in each number of this Review—and such management might please those Athenians who lounge about the medical booksellers' shops, or dip into periodicals for something to *amuse* a dull hour. But what would our sober plodding country and colonial readers say to this, who expect articles which they are to peruse slowly and attentively, and preserve for *re-perusal* and reference in the intervals of their practice? We know well what they would say; and we shall give them no cause for complaint on this head. They may rely on it, that not even Atalanta's *golden* apples shall entice us from the firm and straight forward path on which we have so far trodden with safety to ourselves, and advantage to them. We grant, indeed, that it is both necessary and proper for some people to—" chronicle small beer"—because there are appetites for every species of food, and some prefer " soup meagre" or " Bologna sausages," to roast beef and plumb pudding. Every literary caterer therefore is right in providing for the particular goût of his customers.

---

